# Concussion Reporting Expectation Screening in the Preparticipation Physical Examination

**DOI:** 10.1177/19417381251372975

**Published:** 2025-09-22

**Authors:** Alexandra Abbott, Alexandra M. Klomhaus, Aurelia Nattiv, Joshua Goldman

**Affiliations:** † Division of Sports Medicine, University of California - Los Angeles, Los Angeles, California; ‡ Department of Medicine Statistics Core, David Geffen School of Medicine, University of California - Los Angeles, Los Angeles, California; § Department of Orthopaedic Surgery, Division of Sports Medicine and Nonoperative Orthopaedics, University of California—Los Angeles, Los Angeles, California; ‖ Department of Family Medicine, Division of Sports Medicine, University of California - Los Angeles, Los Angeles, CA

**Keywords:** clinical assessment/grading scales, general sports trauma, head injuries/concussion, injury prevention

## Abstract

**Background:**

Studies of National Collegiate Athletic Association (NCAA) athletes have been concerning for rates of athletes reporting playing while symptomatic with a concussion and of athlete nondisclosure of concussion symptoms.

**Purpose/Hypothesis:**

This study’s primary aim was to determine whether application of a brief and validated scale for concussion reporting expectation (CR-E) is an effective screening tool for collegiate athletes. It was hypothesized that 20% of athletes would be considered high risk for nondisclosure of concussion symptoms using this screening tool.

**Study Design:**

Cross-sectional.

**Level of Evidence:**

Level 2B.

**Methods:**

NCAA Division I athletes (*n* = 358) from 18 teams who presented for preparticipation physical examinations (PPEs) in the 2023 fall season were queried with the CR-E and concussion history questions. The time to complete the CR-E was recorded, and providers recorded whether the screening results warranted additional concussion counseling or education. We report survey responses for all athletes stratified by sex and sport. Power analysis study population was based on NCAA collegiate athletes.

**Results:**

The CR-E required an average of 2 minutes for athletes to complete during their PPE. Of 238 of 358 athletes who completed the CR-E questionnaire, 2 in 3 were determined to benefit from concussion education. Overall, athletes felt “neutral” about their agreement on a Likert scale to report concussion symptoms in 4 scenarios described on the CR-E. Water polo, volleyball, women’s soccer, gymnastics, and football represented sports with higher rates of counseling, below average agreement to report concussion symptoms for 4 scenarios queried, and most frequent nonreporting histories.

**Conclusion:**

The CR-E questionnaire represents an impactful screening tool with high utility and feasibility for collegiate athletes.

**Clinical Relevance:**

Our study supports a brief screening tool to guide concussion counseling for athletes. Based on our data, there appears to be room for improvement in athlete concussion education overall.

In all States in the United States, there are concussion and return to play laws or guidelines for athletes diagnosed with concussion.^23^ At the high school and collegiate levels, concussion education is often mandatory before sport participation. As legislation has been developed recently to require concussion education for athletes, efficacy of curricula is often based on knowledge outcomes.^1,9^ Critique of the pre- and post-test model for testing athletes’ knowledge related to concussion before and after education program completion has prompted support for emphasis for behavior-based outcomes to measure education efficacy instead.^1,5^

Studies of youth and collegiate athletes have demonstrated association between concussion education and concussion knowledge,^5,6,8,23^ but not an association between education or knowledge with reporting behaviors or intention to disclose symptoms. In a study of collegiate athletes by Carroll-Alfano,^8^ knowledge improvements were apparent after concussion education; however, athletes were just as unlikely to report concussion symptoms as those who did not receive concussion education. A study of National Collegiate Athletic Association (NCAA) women’s ice hockey players by Piana et al,^23^ and a study of 741 NCAA athletes by Anderson et al^2^ both demonstrated the concerning inverse relationship between concussion knowledge measures and measures of symptom self-reporting behaviors.

Diagnosis of concussion is often followed by a protocolized return to play progression. Progressions and assessment tools have been updated frequently recently, as concussion research provides new, more effective, and more specific concussion measurement modalities. Providers can make concussion diagnoses based on objective findings such as physical examination, evidence of symptoms without patient endorsement, cognitive testing, balance testing, vestibular-ocular-motor testing, and King-Devick testing.^4,11,12,20,21^ However, the initial diagnosis is often supported or made solely by athletes’ endorsement of symptoms in the setting of a recent head trauma, and return to play is typically guided by athletes’ report of symptom progression.^7,30^

In 2021, Kroshus et al^15,19^ created the concussion reporting expectation questionnaire (CR-E) to improve assessment of planned and reactive decision-making regarding athlete self-report of concussive symptoms. The CR-E was validated with cognitive interviews in youth athletes aged 9 to 16 years, with a final questionnaire demonstrating application to athletes from team and individual sports and to athletes in varying levels of elite status. For example, the initial survey items “. . .even though I am a top player” and “. . .even if my team is losing” were found to not resonate or apply to athletes from different sports or team cultures, and modified or omitted based on athlete feedback. Another question item assuming athletes’ understanding of the definition of a concussion was modified to describe “feeling dizzy after a bump or hit to the head” to address athlete feedback regarding limitations in medical literacy.

Our study aimed to determine whether application of the CR-E during preparticipation physical examinations (PPEs) would result in identification of collegiate athletes who may benefit from additional counseling or education regarding concussion by their provider. High utility based on impactful risk stratification and high feasibility based on survey length and patient accessibility are also important measures to consider for useful screening tool application.

## Methods

**Figure 1.**
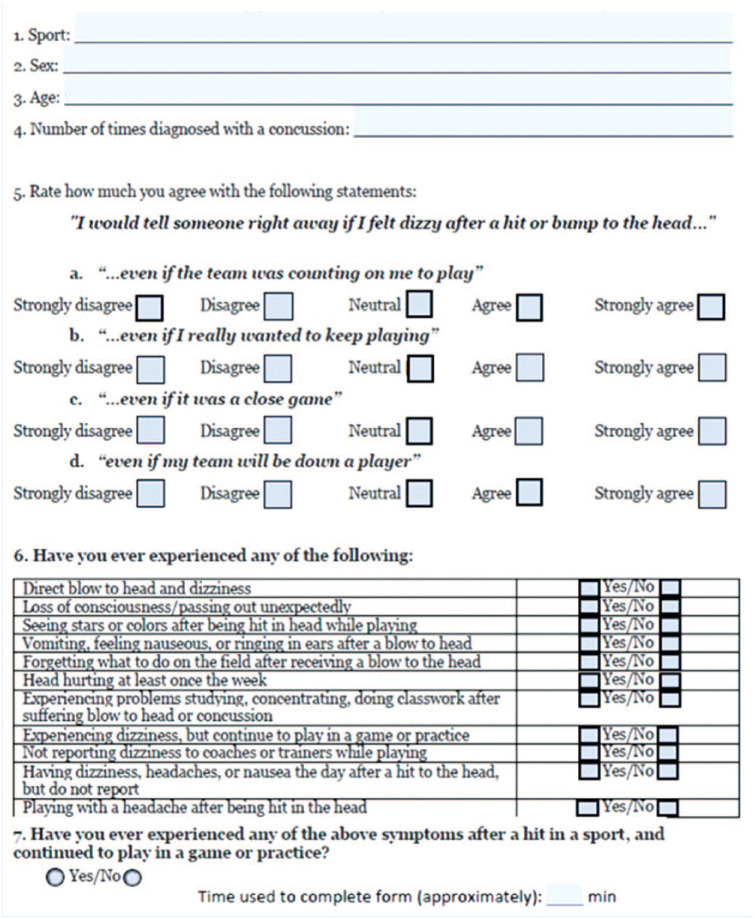
CR-E and additional concussion history questions. CR-E, concussion reporting expectation questionnaire.

In Fall 2023, the CR-E questionnaire was offered on paper or electronically to all athletes in our National Collegiate Athletic Association (NCAA) Division I institution who presented for PPEs with sports medicine physicians (Figure 1). The CR-E and additional concussion questions were queried in addition to existing screening and medical history forms. To ensure awareness of optional research participation, the CR-E was not included with a packet of required forms but instead provided individually to consenting athletes The time to complete the CR-E and concussion history questions was recorded. Providers reviewed the athletes’ CR-E forms and recorded whether their responses warranted additional concussion counseling or education based on the screening. Providers’ review of CR-E responses and decision to provide additional counseling was at the discretion of the provider conducting the athlete’s PPE. This study received an IRB approval from the University of California—Los Angeles.

We present descriptive statistics, including means and standard deviations for continuous variables and counts and frequencies for categorical variables. Categorical CR-E variables include whether counseling/education is warranted and an athlete’s history of continuing to play with concussion symptoms. Continuous variables included the response for “number of previous concussions.” Individual responses to the level of agreement to report a suspected concussion for various scenarios (a Likert scale response) was also considered a continuous variable. Suspected concussion, described as “feeling dizzy after a hit or bump to the head” was based on the definition from the CR-E, validated in youth athletes.^19^ Athletes were queried about specific concussion symptoms, utilizing a survey previously described for assessing prevalence of head injuries and related symptoms in collegiate athletes.^22^ The “history of concussion symptom score” as a continuous variable summates the total number of affirmative responses regarding history of experiencing 11 specific concussion symptoms. This is irrespective of number of previous concussions; ie, if an athlete had several concussions but had experienced only one of the symptoms for both, their symptom score would be 1. With this methodology, symptom history is unique as a variable and not redundant with an athlete’s number of previous concussions variable. We further stratify CR-E responses by athlete sex and sport. To evaluate internal consistency of the CR-E items on a collegiate sample, we calculated Cronbach’s alpha values, both overall and stratified by sex and sport. We used nonparametric tests, including the Wilcoxon rank-sum test (and the Kruskal-Wallis test, when applicable), and chi-square tests to compare differences between subgroups on continuous and categorical variables, respectively. Nonparametric tests were selected for continuous variables due to the distribution of survey responses. All statistical analyses were conducted using SAS Version 9.4 (SAS Institute).

## Results

Of 679 athletes from 18 athletic teams at our institution, 358 (53%) completed the survey; 62% of participants were women and 38% were men. The survey required 1.95 minutes for completion time on average (SD, 1.52), with a median completion time of 2.0 minutes (interquartile range [IQR], 1.0). When stratified by sport and sex, there were high internal consistency scores for survey items (Cronbach alpha ≥ 0.8). The overall Cronbach alpha, as well the Cronbach alpha among male and female athletes, was 0.95. By-sport Cronbach alpha values were as follows: baseball α = 0.95, beach volleyball α = 0.98, football α = 0.98, golf α = 0.92, gymnastics α = 0.80, rowing α = 0.95, soccer α = 0.94, softball α = 0.97, swimming α = 0.97, tennis α = 0.94, track α = 0.97, volleyball α = 0.94, and water polo α = 0.93.

Of the 358 athletes who completed the CR-E, 238 (66%) warranted additional counseling based on their responses. Athletes from gymnastics, women’s volleyball, and women’s water polo demonstrated the highest indication for provider counseling or education regarding concussion (Table 1). Athletes from all represented sports demonstrated counseling needs ≥50%.

**Table 1.** Proportion of athletes determined to benefit from additional counseling based on CR-E review

	Number of respondents	Counseling/education warranted
All athletes	358	238 (66%)
Sex		
Female	222	148 (67%)
Male	136	90 (66%)
Sport		
Gymnastics	10	9 (90%)
Women’s volleyball	6	5 (83%)
Women’s water polo	25	19 (76%)
Men’s volleyball	18	14 (78%)
Softball	17	13 (76%)
Women’s tennis	8	6 (75%)
Men’s water polo	7	5 (71%)
Men’s golf	17	12 (71%)
Football	20	14 (70%)
Men’s soccer	13	9 (69%)
Women’s soccer	3	2 (67%)
Women’s beach volleyball	17	11 (65%)
Women’s rowing	65	42 (65%)
Baseball	32	20 (63%)
Men’s track and field, cross country	25	15 (60%)
Women’s track and field, cross country	40	24 (60%)
Men’s tennis	9	5 (56%)
Women’s swim and dive	26	13 (50%)

CR-E, concussion reporting expectation questionnaire.

A total of 67 (19%) of the 358 responding athletes endorsed a history of continuing to play without reporting concussion symptoms (Table 2). Overall, the average number of previous concussion diagnoses for all athletes was 0.36 (SD, 0.80) (Table 3); however, the median number of diagnoses was 0 (IQR, 0). The average number of previous concussion symptoms after a hit to the head in athletes from all sports was 1.37 (SD, 2.35), corresponding to a median of 0.0 and an IQR of 2.0, out of 11 suggested symptoms. Athletes from men’s water polo, women’s soccer, and gymnastics were the most likely athletes to report a history of a concussion diagnosis. Athletes from men and women’s water polo, women’s soccer, and football reported the greatest history of concussion symptoms.

**Table 2.** Proportions of athletes reporting previously continuing to play with concussion symptoms

	History of continuing to play with concussion symptoms n (%)
All athletes	67 (19)
Sex	
Female	40 (18)
Male	27 (20)
Sport	
Women’s soccer	2 (67)
Women’s water polo	11 (44)
Men’s water polo	3 (43)
Football	8 (40)
Gymnastics	4 (40)
Men’s volleyball	6 (33)
Women’s volleyball	2 (33)
Women’s beach volleyball	4 (24)
Men’s soccer	3 (23)
Women’s track/cross country	5 (13)
Men’s track/cross country	3 (12)
Women’s rowing	8 (12)
Softball	2 (12)
Men’s tennis	1 (11)
Baseball	3 (9)
Women’s swim and dive	2 (8)
Women’s tennis	0 (0)
Men’s golf	0 (0)

**Table 3.** Athletes’ report of previous concussion diagnoses and previous history of concussion symptoms after a hit in sport

	Number of previous concussions Mean (SD) / Median (IQR)	History of symptom score Mean (SD) / Median (IQR)
All athletes	0.36 (0.80) / 0.00 (0.00)	1.37 (2.35) / 0.00 (2.00)
Sex		
Female	0.37 (0.84) / 0.00 (0.00)	1.41 (2.39) / 0.00 (2.00)
Male	0.34 (0.72) / 0.00 (0.00)	1.28 (2.28) / 0.00 (2.00)
Sport specific		
Men’s water polo	1.00 (1.53) / 0.00 (2.00)	3.00 (3.61) / 3.00 (4.00)
Women’s soccer	1.00 (1.73) / 0.00 (3.00)	2.67 (3.06) / 2.00 (6.00)
Gymnastics	1.00 (1.15) / 0.50 (2.00)	1.80 (2.62) / 0.50 (3.00)
Women’s volleyball	0.83 (2.04) / 0.00 (0.00)	1.33 (2.42) / 0.00 (2.00)
Men’s volleyball	0.78 (1.11) / 0.00 (1.00)	2.06 (3.11) / 0.50 (3.00)
Softball	0.76 (1.30) / 0.00 (1.00)	1.71 (1.65) / 2.00 (3.00)
Women’s water polo	0.40 (0.76) / 0.00 (1.00)	2.80 (3.03) / 1.00 (6.00)
Football	0.30 (0.47) / 0.00 (1.00)	2.15 (2.72) / 1.00 (4.00)
Women’s rowing	0.29 (0.73) / 0.00 (0.00)	1.29 (2.55) / 0.00 (1.00)
Women’s beach volleyball	0.29 (0.47) / 0.00 (1.00)	1.12 (1.62) / 0.00 (1.00)
Men’s track/cross country	0.29 (0.62) / 0.00 (0.00)	1.00 (1.96) / 0.00 (1.00)
Women’s track/cross country	0.28 (0.68) / 0.00 (0.00)	1.13 (2.22) / 0.00 (1.00)
Women’s swim and dive	0.27 (0.60) / 0.00 (0.00)	1.27 (2.46) / 0.00 (1.00)
Men’s soccer	0.23 (0.44) / 0.00 (0.00)	0.62 (1.45) / 0.00 (0.00)
Baseball	0.19 (0.40) / 0.00 (0.00)	0.70 (1.49) / 0.00 (1.00)
Men’s tennis	0.13 (0.35) / 0.00 (0.00)	0.22 (0.44) / 0.00 (0.00)
Men’s golf	0.12 (0.49) / 0.00 (0.00)	0.88 (1.87) / 0.00 (0.00)
Women’s tennis	0.00 (0.00) / 0.00 (0.00)	0.00 (0.00) / 0.00 (0.00)

On average, athletes reported “neutral” agreement on a 1 to 5 Likert scale to report suspected concussion immediately for various proposed scenarios on the CR-E (Table 4). These included “if the team was counting on me to play” (average score 3.5 out of 5), “if I wanted to keep playing” (3.5), “if it was a close game” (3.4), and “if the team is down a player” (3.3). Comparisons between male and female athletes demonstrated no significant difference in the number of times previously diagnosed with a concussion (*P* = 0.76), intention to continue to play with a suspected concussion for various proposed scenarios (continuing to play if team was counting on them (*P* = 0.53), wanting to keep playing (*P* = 0.35), close game (*P* = 0.12), down a player (*P* = 0.46)), the history of symptom score (*P* = 0.53), whether they continued to play with symptoms (*P* = 0.62), and whether CR-E screening warranted additional counseling by the reviewing provider (*P* = 0.88). Corresponding descriptive statistics stratified by male/female athletes can be found in Tables 1 to 4.

**Table 4.** Athletes’ average reports of agreeing to report concussion symptoms in various sport scenarios

	". . .team counting on me" Mean (SD) / Median (IQR)	". . .I wanted to keep playing" Mean (SD) / Median (IQR)	". . .close game" Mean (SD) / Median (IQR)	". . .down a player" Mean (SD) / Median (IQR)
All athletes	3.51 (1.21) / 4.00 (1.00)	3.49 (1.16) / 4.00 (1.00)	3.35 (1.22) / 3.00 (2.00)	3.27 (1.24) / 3.00 (2.00)
Sex				
Female	3.54 (1.20) / 4.00 (1.00)	3.53 (1.14) / 4.00 (1.00)	3.43 (1.19) / 4.00 (1.00)	3.31 (1.22) / 3.00 (2.00)
Male	3.46 (1.22) / 4.00 (1.50)	3.41 (1.20) / 4.00 (2.00)	3.22 (1.26) / 3.00 (2.00)	3.20 (1.28) / 3.00 (2.00)
Sport				
Baseball	3.38 (1.36) / 3.00 (2.50)	3.38 (1.24) / 4.00 (1.50)	3.25 (1.30) / 3.00 (2.00)	3.06 (1.29) / 3.00 (2.00)
Women’s beach volleyball	3.47 (1.33) / 4.00 (1.00)	3.65 (1.06) / 4.00 (1.00)	3.35 (1.27) / 4.00 (1.00)	3.24 (1.35) / 4.00 (2.00)
Football	3.15 (1.42) / 3.00 (2.50)	3.10 (1.37) / 2.50 (2.50)	3.15 (1.35) / 3.00 (2.50)	3.15 (1.31) / 3.00 (2.50)
Men’s golf	3.65 (1.11) / 4.00 (1.00)	3.18 (1.19) / 3.00 (2.00)	2.82 (1.33) / 3.00 (2.00)	3.18 (1.19) / 3.00 (1.00)
Gymnastics	3.40 (1.17) / 3.00 (1.00)	2.90 (0.99) / 3.00 (2.00)	2.60 (0.97) / 2.50 (1.00)	2.90 (1.20) / 3.00 (2.00)
Women’s rowing	3.61 (1.15) / 4.00 (1.00)	3.66 (1.07) / 4.00 (1.00)	3.58 (1.08) / 4.00 (1.00)	3.36 (1.13) / 3.00 (1.00)
Men’s soccer	3.23 (1.01) / 3.00 (1.00)	3.31 (1.38) / 3.00 (1.00)	3.23 (1.30) / 3.00 (2.00)	2.92 (1.50) / 3.00 (2.00)
Women’s soccer	3.33 (1.15) / 4.00 (2.00)	3.00 (1.00) / 3.00 (2.00)	3.00 (1.00) / 3.00 (2.00)	2.33 (1.53) / 2.00 (3.00)
Softball	3.53 (1.33) / 4.00 (1.00)	3.47 (1.23) / 4.00 (1.00)	3.24 (1.25) / 3.00 (1.00)	3.24 (1.30) / 3.00 (2.00)
Women’s swim and dive	3.92 (1.26) / 4.00 (2.00)	4.04 (1.11) / 4.00 (2.00)	4.00 (1.20) / 4.50 (2.00)	4.08 (1.16) / 5.00 (2.00)
Men’s tennis	4.00 (1.12) / 4.00 (2.00)	3.89 (1.27) / 4.00 (2.00)	3.56 (1.42) / 4.00 (2.00)	3.22 (1.39) / 3.00 (2.00)
Women’s tennis	3.63 (1.60) / 4.00 (2.50)	3.75 (1.39) / 4.00 (2.00)	3.88 (1.36) / 4.00 (1.50)	3.38 (1.30) / 3.00 (1.50)
Men’s track, cross country	3.64 (1.25) / 4.00 (2.00)	3.60 (1.12) / 4.00 (1.00)	3.36 (1.22) / 3.00 (1.00)	3.40 (1.26) / 4.00 (2.00)
Women’s track, cross country	3.63 (1.17) / 4.00 (1.50)	3.50 (1.26) / 4.00 (1.50)	3.53 (1.28) / 4.00 (2.50)	3.40 (1.28) / 4.00 (1.50)
Men’s volleyball	3.33 (1.08) / 3.00 (1.00)	3.33 (1.03) / 3.00 (1.00)	2.94 (1.11) / 3.00 (2.00)	3.11 (1.28) / 3.00 (2.00)
Women’s volleyball	2.83 (1.17) / 2.50 (1.00)	3.17 (1.17) / 3.00 (2.00)	3.00 (1.55) / 3.00 (2.00)	2.83 (1.17) / 2.50 (1.00)
Men’s water polo	3.57 (0.98) / 4.00 (1.00)	3.57 (0.98) / 4.00 (1.00)	3.57 (0.98) / 4.00 (1.00)	3.71 (0.95) / 4.00 (1.00)
Women’s water polo	3.16 (0.99) / 3.00 (2.00)	3.24 (0.93) / 3.00 (1.00)	3.04 (0.89) / 3.00 (2.00)	2.80 (0.96) / 3.00 (1.00)

## Discussion

Overall, the CR-E demonstrated feasibility, utility, and internal consistency when applied to NCAA Division I collegiate athletes in this large and diverse cohort. Its ease of use and accessibility is demonstrated by the engagement of >50% of the collegiate athlete population at our institution for an optional survey without additional incentive, and the average completion time of 2 minutes for the survey. Comparative values between male and female athletes and between athletes from different sports, as well as high Cronbach alpha scores for question items support internal consistency and appropriate application of the CR-E to collegiate athletes. Finally, the identification of 2 in 3 athletes who may benefit from additional discussion about concussion reporting by their physicians in the PPE setting demonstrates the high yield and potential impact for this screening tool in collegiate athletes.

A study of collegiate athletes by Kroshus and Baugh^14^ found that, although 91% of athletes received concussion education primarily from athletic trainers, 55% indicated that they would like education delivered by a physician. A survey used as a screening tool can allow physicians to identify opportunities to supplement athletes’ education and potentially improve reporting behaviors for future concussions. In the preparticipation setting, before active concern for concussion, planned and reactive decision-making can be addressed in an objective, confidential physician-patient discussion. It will be important in future research to determine the effects of physician counseling on reporting behaviors, especially when compared with current concussion education programming that does not emphasize physician involvement. It is optimistic that a tool like the CR-E can serve as a means to identify athletes who may benefit from additional counseling, and that athletes are indicating that they would like more education from physicians specifically.

Overall, athletes in our cohort felt “neutral” regarding agreement to report concussion symptoms in various sport scenarios—the average agreement levels were approximately 3 out of 5. Providers who identified low agreement scores possibly stratified athletes to benefit from further counseling. Consequently, the average lack of agreement scores alone possibly contributed to the high frequency of athletes identified to benefit from additional concussion discussion, despite other items on the survey being considered

However, other aspects of athletes’ answers were also concerning and indicative of opportunities to improve overall concussion education and reporting behaviors. In our study, 19% of athletes endorsed previously continuing to play without reporting known concussion symptoms. Of note, history of nonreporting, based on the survey by Kaut et al,^22^ does not discriminate whether athletes suspected concussion at the time of their symptoms. However, of responding athletes, 67% of athletes from women’s soccer, 44% of water polo athletes, 40% of football players, and 40% of gymnasts reported continuing to play in a game or practice with suspected concussion after a hit in sport. These results are concordant with a 2020 study of NCAA student-athletes by Stamm et al^29^ demonstrating that, of athletes with history of concussion, 20% never disclosed their symptoms and 68% reported continued competitive sport participation while symptomatic.

In screening athletes from our large institution, we achieved a secondary aim of contributing epidemiological data regarding sport- and sex-specific concussion reporting expectation in collegiate athletes. Overall, male and female athletes did not differ significantly when answering questions about their history of concussion diagnoses or concussion symptoms, previously continuing to play with concussion symptoms, or their agreement to report concussion symptoms for a variety of scenarios. On average, providers determined the same rates of counseling needs for male and female athletes (66%) as well.

However, specific comparisons of sports with men’s and women’s teams demonstrated some differences in reporting behaviors despite playing the same sport. Athletes from women’s soccer, men and women’s water polo, football, and gymnastics were the most likely to endorse a history of continuing to play in a game or practice without reporting concussion symptoms. Notably, women’s soccer players endorsed this 67% of the time, whereas men’s soccer players indicated this history 23% of the time. This may be related to increased concussion incidence found in female soccer players when compared with male soccer players at the youth and collegiate levels.^3,10,24^ This disparity in concussion incidence is corroborated in our collegiate cohort, with male soccer players reporting fewer historical concussions than female soccer players (0.23 and 1 on average, respectively). However, the disparity in reporting behavior with an inverse relationship to typical concussion incidence may indicate differences in social or cultural norms when comparing men’s and women’s soccer. Although both teams represent athletes of elite status, the women’s soccer team at our institution is recently in contention for championship status. Differences in level of competition between the men’s and women’s soccer teams also may contribute to different social or cultural influences. Further research may elucidate relationships between level of play, prospective concussion incidence, and reporting behaviors across different teams.

Understanding social factors and external pressures affecting athletes is important for identifying opportunities to influence athletes’ willingness to report symptoms. Register-Mihalik et al^27^ studied 281 first-year NCAA student-athletes to associate potential determinants of concussion disclosure behaviors. During preseason evaluation, a validated survey included questions regarding concussion history and disclosure, attitudes and perceived social norms, intention to disclose, and control over concussion disclosure. Social norms encompassed organizational and peer perceptions of the athlete and their expected behaviors. They demonstrated a consistent relationship between perceived positive social norms and disclosure behaviors. They specifically demonstrated a significantly positive association between athletes reporting never having participated in sport while with concussion symptoms (*P* = 0.02, prevalence ratio 1.50) and intention to disclose concussion symptoms (*P* < 0.001, prevalence ratio 1.34) with more positive perceived norms scoring.^27^ These authors, as well as recent and concordant studies, have concluded that improving misperceived or actual social norms supports improved concussion disclosure.^16-18,25,26,28^ In an organization with multidisciplinary stakeholders, this includes educational efforts for the athletes but also includes healthcare providers, athletic trainers, coaches, parents, and fans.

In our study, athletes from men’s water polo, women’s soccer, and gymnastics endorsed the greatest concussion history. Men and women’s volleyball, softball, and women’s water polo also demonstrated the greatest number of previous concussions when compared with the overall athlete cohort. Athletes from men and women’s water polo, women’s soccer, football, and gymnastics reported the highest number of historical concussion symptoms. Football players reported an average of 0.30 for the number of previous concussions. Despite this indicating that the majority of athletes did not report a previous concussion, football players were among athletes with the highest number of concussion symptoms after a hit to the head in sport. This discrepancy may reflect missed diagnoses in the past, a need for education regarding the definition of concussion, or that football players with concussion diagnoses had more symptoms on average than athletes with concussion diagnoses in other sports. The discrepancy between number of previous concussion diagnoses and the number of historical concussion symptoms was also apparent in nearly every other sport in our study, with the exception of men and women’s tennis, who reported minimal or no history of concussion diagnoses or symptoms.

Athletes’ rates of reporting previous concussion diagnoses are somewhat concordant with studies of collegiate athlete concussion incidence by sport in the current study. Sports with the highest reported history of concussion or symptoms were men and women’s water polo, women’s soccer, gymnastics, men and women’s volleyball, softball, and football. A 16-year epidemiological study of 15 NCAA sports by Hootman et al^13^ found that football, men’s and women’s ice hockey, and women’s soccer represented the sports with above athlete average concussion incidence rates. A similarly modeled study of 25 NCAA sports by Zuckerman et al^31^ demonstrated women’s lacrosse, women’s basketball, women’s soccer, football, men’s and women’s ice hockey, and wrestling to be above average for concussion incidences compared with athletes in other sports. Ice hockey, lacrosse, and wrestling are not offered at our institution for comparison. Gymnastics has demonstrated apparently relatively low concussion incidence in studies like these that include diagnoses that require medical attention and sport restriction. However, in this study measuring self-reported previous or resolved concussions that do not prompt current evaluation, gymnasts reported greater histories than athletes from nearly every other sport.

Sports with athletes least likely to report suspected concussion when compared with athletes’ average scores overall were baseball, football, golf, gymnastics, soccer, volleyball, and water polo. Overall, it appears that positive reporting intention from athletes from beach volleyball, rowing, softball, swim and dive, tennis, and track and field/cross country offset poorer reporting intention in the sports with below average reporting expectation scores. This resulted in an overall neutral athlete agreement in our cohort.

The athletes with the highest counseling benefit determined by their providers were in gymnastics (90% of athletes), women’s volleyball (83%), and women’s water polo (79%). However, athletes from each and every sport examined warranted counseling at proportions of 50% or higher.

**Figure 2.**
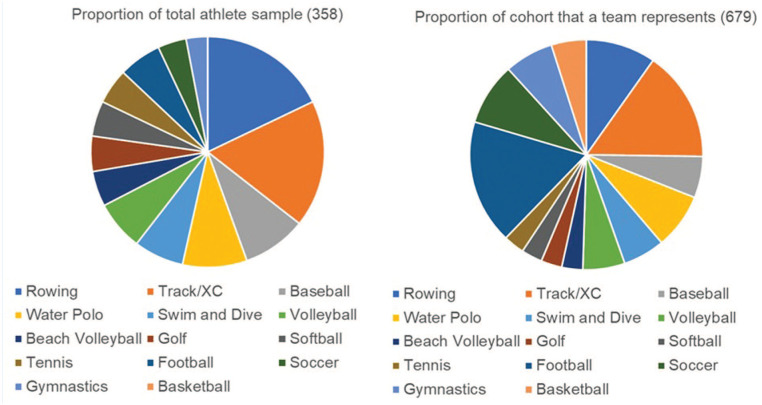
Comparative representation of athletes in sample versus athletes in cohort to indicate specific sport representation. XC, cross country.

Limitations of our study include apparent sampling and response bias, with female athletes electing to participate in the survey more frequently than male athletes; some sports were not as well represented by the athletes who chose to participate (Figure 2 and Table 5). Athletes from basketball, football, gymnastics, and soccer were the least proportionately represented based on the sizes of their teams and overall athlete cohort. Notably, these sports are scrutinized and studied frequently for concussion associations. Athletes who elected not to engage in the study may represent those who would have completed the CR-E differently than those who did participate. Finally, though our cohort is large and survey engagement was adequate, there is limited generalizability by subgroup such as sex and sport. Each sport only contributes a sample size of 10 to 65 per group based on team sizes. However, collegiate and elite athlete generalizability is conceivable with our data.

**Table 5.** Comparative representation of athletes in sample versus athletes in cohort to indicate specific sport representation

Sport	Number of respondents, proportion of total athletes N (%)	Proportion of cohort that a team represents (n = 679)
Rowing	65 (18)	10%
Track and field, cross country	65 (18)	16%
Baseball	32 (9)	6%
Water polo	32 (9)	8%
Swim and dive	26 (7)	6%
Volleyball	24 (7)	6%
Beach volleyball	17 (5)	3%
Golf	17 (5)	3%
Softball	17 (5)	3%
Tennis	17 (5)	3%
Football	20 (6)	18%
Soccer	16 (4)	9%
Gymnastics	10 (3)	7%
Basketball	0 (0)	5%

Regarding provider-specific limitations, the CR-E does not come with a designated points system for interpretation. Providers were tasked to determine individually how much disagreement to report symptoms would warrant additional counseling. Future modifications of the CR-E may benefit from validation of an interpretation scale for risk assessment, similarly to those used for mood screening such as the Patient Health Questionnaire or Generalized Anxiety Screening. Given the lack of validation and risk association for CR-E responses currently, providers determined at their discretion whether disagreement responses warranted preseason counseling for each athlete. Although this presents a possible inter-rater difference, it may better represent real-world application; physicians frequently utilize their gestalt when reviewing screening questionnaires to determine whether responses appear high risk for their patients.

Our study demonstrates that the CR-E may contribute impactful and accessible screening in the preparticipation exam setting. Overall, it is apparent that there is significant room for improvement and opportunity to address gaps in concussion education programs for collegiate athletes. The high rates of history of nonreporting, lack of agreement to report future symptoms, and discrepancies in surveys indicating potential missed diagnoses or misunderstanding of concussion symptoms are resounding. Lack of intention to report symptoms represents a barrier to initial diagnosis, safe return to play progression, and prevention of prolonged recovery time. Based on our findings, future research directions may include the effect of physician counseling on concussion incidence in athletes. It will also be important to continue developing and validating questionnaires like the CR-E to be used for screening and concussion education program assessment for various athlete populations.
